# A Universal Mental Health Promotion Programme for Young People in Italy

**DOI:** 10.1155/2015/345926

**Published:** 2015-08-25

**Authors:** Antonella Gigantesco, Debora Del Re, Isabella Cascavilla, Gabriella Palumbo, Barbara De Mei, Chiara Cattaneo, Ilaria Giovannelli, Antonino Bella

**Affiliations:** National Centre of Epidemiology, Surveillance, and Health Promotion, Italian National Institute of Health, Viale Regina Elena 299, 00161 Rome, Italy

## Abstract

In Italy, the Mental Health Unit of the National Institute of Public Health has developed a school-based mental health programme based on a structured handbook. The aim of this programme is to promote self-efficacy, psychological well-being, and life satisfaction. In this study, we evaluated the effectiveness of this programme. We used data from 308 students who participated in a study in 9 Italian high schools during the 2011-2012 school year. In order to analyse the school intervention programme, we set up a pre-post test design study involving 18 classrooms (8 of which acting as a control). The schools were selected via a snowball technique, and then the classrooms that agreed to participate were randomly assigned to intervention and control groups. The programme was performed during regular school hours in one-hour a week sessions for a total of 20 hours of classroom time. Assessments before and 2 months after the programme were performed using Regulatory Emotional Self-Efficacy, Satisfaction With Life, and Ryff's Psychological Well-Being Scales. The results showed an improvement in self-efficacy in regulating negative affect, overall psychological well-being, and satisfaction with life. These results demonstrate that the programme produced significant positive effects on the mental health status of participating students.

## 1. Introduction

There is growing concern in our country regarding the increasing number of children and adolescents that are having difficulty in resolving the problems that arise during their development. According to the fifth and sixth reports on youth [1, 2] carried out by the IARD Institute, a nonprofit organization in Milan involved in sociological research, an increasing number of young people suffer from apathy, sadness, and low self-esteem and seem to lack the capacity to define long-term goals and life choices. At the same time, many adolescents have internalizing problems such as anxiety and depression. According to the PRISMA study (a national project on children's mental health), which represents the only investigation conducted in Italy aimed at evaluating the prevalence of mental disorders among children aged 10–14 years, 8.2% (CI 4.2%–12.3%) of preadolescents were found to suffer from at least one externalizing or internalizing mental disorder.

Poor mental health in childhood and adolescence is associated with health and social problems such as school failure, delinquency, and substance misuse, and this increases the risk of adverse outcomes in adulthood [3]. Interventions that promote positive mental health may provide young people with the necessary life skills, support, and resources to fulfil their potential and overcome adversity. The most important life skills include self-efficacy, problem-solving, empathy, and coping strategies [4].

Schools represent one of the most important community settings where the mental health of young people can be promoted because they are sources of a significant number of young people who experience mental health problems [5]. In fact, the promotion of emotional health and well-being is a core feature of the WHO's Health Promoting Schools initiative [6]. Reviews of the literature suggest that mental health promotion programmes in schools, especially those adopting a wider approach, namely, the ones that promote generic psychosocial competence instead of focusing on specific behavioural problems, produce long-term benefits for young people, including improved emotional and social functioning and positive health behaviour [7].

In 2009, the Mental Health Unit (MHU) of the* Istituto Superiore di Sanità* (ISS) (Italy's National Institute of Public Health) obtained a grant from the Ministry of Health to develop a school-based programme in order to promote mental health. The MHU has developed a programme designed to reach all students (universal programme), regardless of their level of risk regarding the development of a mental or behavioural problem. Stronger effects are found in targeted prevention compared to universal prevention, but in targeted prevention not all adolescents at risk are identified [8]. In universal prevention, the stigmatization effect associated with targeted prevention is no longer an issue [9], and all adolescents can be involved.

Adolescence is a period in which emotional intelligence and self-efficacy beliefs are malleable and this is important given that these variables are strongly related to depression and related disorders [10, 11].

Emotional intelligence [12] refers to individual differences in the perception, processing, regulation, and utilization of emotional information. Individuals with high emotional intelligence believe that they are in touch with their emotions and can regulate them in a way that promotes well-being [13] and life satisfaction [14].

Self-efficacy aims at a broad and stable sense of personal competence which can deal effectively with a variety of stressful situations [15]. In fact, self-efficacy determines an individual's resilience to adversity and his/her vulnerability to stress and depression [16].

It is therefore important to address emotional intelligence and self-efficacy in enhancing the positive development of the young people because they can promote psychological well-being and life satisfaction, which in turn could decrease the incidence of internalizing disorders [17].

Our programme was developed for secondary schools in order to promote or optimize self-efficacy, psychological well-being, and satisfaction with life, which are fundamental components of mental health status [17–19]. The programme combines training regarding problem-solving and the development of emotional intelligence skills in order to better deal with the demands of everyday life and to cope effectively with life stress. It also provides information about the nature of major mental disorders. It was inspired by the approach adopted by Falloon in the psychosocial rehabilitation for caregiver-based stress management of schizophrenic and affective disorders [20].

In order to determine whether or not the programme was effective, in the 2010-2011 school year, we performed a pre-post test study design with a control group in 9 Italian high schools. The short-term results are reported in the present paper. We hypothesized that the programme would have led to higher self-efficacy, psychological well-being, and satisfaction with life.

## 2. Materials and Methods

### 2.1. Programme

The programme, named* Definizione di obiettivi e soluzione di problemi* (establishing goals and problems solving), was designed for students of between 14 and 18 years of age (i.e., those in high school) and addressed issues such as promoting or maintaining mental health, recognising signs of mental illness, and decreasing the stigma associated with mental illness.

The programme focused mainly on teaching skills that enable students to cope satisfactorily with stress in their life and was inspired by Goleman's emotional intelligence model [21] and Falloon's psychoeducational approach [22]. Goleman's model identified five domains of emotional intelligence: (i) knowing your emotions; (ii) managing your own emotions; (iii) using emotions to motivate yourself; (iv) recognising the emotions of other people; (v) managing relationships. In Falloon's psychoeducational approach, psychiatric patients and their families are trained to use structured problem-solving to address problems that cause them the most stressful situations in their life and to use their social network to obtain the support of the people who are most willing and able to assist them in resolving problems [22]. As in Falloon's approach, the core component of the programme is training in the form of structured* six-step* problem-solving, which is extremely robust and has been widely used in therapeutic programmes [20, 23, 24]. The problem is stated clearly; all potential solutions are brainstormed and listed; each possible solution is evaluated in terms of its main advantages and disadvantages; the most practical solution is chosen; implementation is carefully planned; and the outcome of the implementation efforts is reviewed and refined until the problem has been resolved. There are a number of differences between the approach used in the present programme and Falloon's approach. First, the present programme places a high level of importance on defining personal global goals and suggests dividing complex goals into specific measurable objectives to be dealt with in sequence. The programme does not describe any techniques in detail and it does not require any specific cognitive-behavioural techniques, although it recommends modelling and role playing. Secondly, the present programme teaches what mental illnesses are (i.e., information on causes, symptoms, prodromal and warning signs, and treatment of mental illnesses) in order to increase awareness and contrast the stigma surrounding these illnesses. The illnesses covered are anxiety disorders, depression, manic-depressive disorder, and schizophrenia, which were chosen because of their prevalence among adolescents and early age of onset.

The programme was designed to be implemented in high schools with the help of a student manual that mainly consists of exercises to be conducted in school and at home in order to encourage students to use the skills and information introduced in each unit of the manual. The main contents of the manual address skills are using structured* six-step* problem-solving, defining personal goals, adopting effective communication skills, using negotiation, coping with stress, coping with anger, and resolving conflict. The manual also teaches the students to recognize and modify negative key thoughts and feelings that precede, accompany, and follow unpleasant emotions. These include attributional styles, that is, how individuals explain to themselves the events of their life; for example, individuals with depression have a negative attributional style, in which they attribute the cause of bad events to themselves (personalization), perceive these events as being permanent (persistence), and engage in negative generalization following unsuccessful experiences (generalization). More details of the manual have been published elsewhere [25]. The manual, which is available on the Ministry of Health website (http://www.ccm-network.it/documenti_Ccm/prg_area5/2005-manuale-scuola-depressione.pdf), is about 200 pages long and contains 18 units (Box 1). Each chapter of the manual is the subject matter of one-work session held in school.

### 2.2. Study Design

The study consisted of a pre-post test design study with two conditions: intervention versus control (curricula as usual). The participating schools were contacted in early 2010 using the “snowball” technique, which is a particular form of chain analysis that seeks constructing a sample of individuals with common characteristics [26]. The first schools contacted served as agents for locating other schools. In fact, they introduced us to new schools and put us in touch with students and teachers. Once contact had been established, we illustrated the programme to students and teachers in each school. Eighteen classrooms of 9 high schools located in a medium-sized town (Velletri) and six cities (Piacenza, Brescia, Crema, Ascoli Piceno, Torino, and Pisa) in central and northern Italy volunteered to participate in the study. The students' socioeconomic level was comparable in all classrooms. The classrooms (not the single students) were randomly assigned to either intervention or control group. Four 9th grade (first year of high school) classes, five 10th grade (second year of high school) classes, and one 11th class (third year of high school) were assigned to the programme group. Two 9th grade classes, five 10th grade classes, and one 11th class were assigned to the control group. Finally, the programme was conducted during the 2010-2011 school year between October 2010 and March 2011 in 10 classrooms while 8 acted as controls. Prior to participation, students' parents signed written informed consent. The programme was held in the classroom during regular school hours and needed one-hour session a week for a total of 20 hours class time. Each session was coordinated by a facilitator, who was the psychologist or pedagogist of the school where the programme was implemented. They were trained by researchers of the ISS Mental Health Unit who also developed the programme. Facilitators, after reading the manual, completed the training through a one-day training session which lasted for 6 hours, where they also received a guide regarding who to implement the programme. The guide provided them with practical information to identify and overcome barriers in order to implement the programme correctly [25]. As the study concerned a psychoeducational intervention, it needed no formal approval by the Ethical Committee of the National Institute of Health, which was nevertheless consulted and gave informal authorization. We also conducted this study according to the international guidelines and ethical codes of the Belmont Report and the Oviedo Convention, and according to paragraph 9, section I of the National Psychological Association ethical code.

### 2.3. Study Population

A preintervention test was carried out in October 2010 before the beginning of the programme. Students attending the classrooms completed some self-administered assessment scales (see later). They had to generate a secret password which had to be memorized and reported on all the scales. At the moment of the preintervention test, there were 391 participating students who had an average age of 15.2 years (SE = 0.05); 154 (39.4%) of them were male, 221 of them (56.5%) attended classrooms assigned to the programme group, and 170 (43.5%) attended classrooms assigned to the control group.

Posttest assessment was carried out in June 2011, 2 months after the programme delivery.

Pre- and postprogramme measures were matched using the self-generated anonymous password, and the records from the students who completed the preintervention could be linked to those generated at the postintervention. Forty-five (20.4%) students in the programme group and 38 (22.4%) in the control group were not present in class when the posttest assessment was administered (2 different occasions were provided). Therefore, we had data from 308 students who completed the postintervention assessment (intervention group *N* = 176; control group *N* = 132).

### 2.4. Measures

The study evaluated changes in self-efficacy in regulating emotions, psychological well-being, and life satisfaction.

The assessment scales which were used included:

- The Regulatory Emotional Self-Efficacy (RESE) scales, an instrument developed to assess perceived self-efficacy in regulating negative emotions (8 items) and in expressing positive emotions (7 items); an example of a negative emotion item is: “To what extent are you able to overcome frustration if other people do not appreciate you as you would like?”; an example of positive emotion item is: “To what extent are you able to express happiness when something nice happens?” [16, 19]. Self-efficacy in regulating negative emotions refers to one's ability to improve negative emotional states once they are aroused in response to adversity or frustrating events and how to avoid being overcome by emotions such as anger, irritation, and discouragement. Self-efficacy in expressing positive emotions refers to one's ability to experience or to allow oneself to express positive emotions, such as joy, enthusiasm, and pride, in response to success or pleasant events, and is related to low levels of externalizing and psychopathic problems [27] as well as to low levels of internalizing problems [28]. For each item, participants rated (ranging from 1 [*not well at all*] to 5 [*very well*]) their ability to manage their emotional life with the RESE. The RESE scales have good psychometric properties [16, 19]. In the present study, the internal consistency of the RESE scales was satisfactory, as reflected by Cronbach's alpha value of 0.74. It was also satisfactory considering separately the two subscales on negative emotions (alpha 0.74) and on positive emotions (alpha 0.83).
- The Satisfaction With Life Scale [29], a five-item scale designed to measure the cognitive component of well-being. For each item, participants rated the extent to which they felt generally satisfied with life on a 7-point rating scale (from 1 = strongly disagree to 7 = strongly agree). An example of an item for this scale is “In most ways, my life is close to my ideal.” Scores range from 5 to 35 with higher scores showing greater life satisfaction. The SWLS has been found to be positively associated with other well-being measures and negatively associated with psychopathology measures [29]. In children and adolescents, higher satisfaction with life has been reported to be associated with fewer symptoms of anxiety and depression, less delinquency and aggression, less internalizing and externalizing behaviour, and increased self-esteem [30]. In the present study, the internal consistency of this scale was very satisfactory, as reflected by Cronbach's alpha value of 0.85.
- Ryff's Scales of Psychological Well-Being (PWBS) [31, 32] include 84 items for six 14-item scales, which are constructed to measure six dimensions of psychological well-being: autonomy, environmental mastery, personal growth, positive relations with others, purpose in life, and self-acceptance. Participants respond using a six-point format from strongly disagree (1) to strongly agree. (6) High scores show high positive self-ratings on the dimension assessed. Psychological well-being (PWB) explicitly concerns the individual's self-realization [33]. In the past decade, many studies have shown that low PWB makes people more vulnerable to mental ill-being. Furthermore, very recent research implies impaired PWB levels in the aetiology of depression [34] and suggests that PWB improvement may have mental health implications. The following are examples of items of the six scales:* autonomy*: “My decisions are not usually influenced by what anyone else is doing”;* environmental mastery*: “I am quite good at managing the responsibilities of my daily life”;* personal growth*: “I think new experiences are important because they question how you think about yourself and the world”;* positive relations with others*: “I know that I can trust my friends and they know they can trust me”;* purpose in life*: “I have a sense of direction and purpose in life”;* self-acceptance*: “I like most aspects of my personality”.
- In the present study, the six subscales yielded acceptable to excellent internal consistency, as reflected by Cronbach's alpha value of 0.81 for autonomy, 0.75 for environmental mastery, 0.68 for personal growth, 0.80 for positive relations with others, 0.76 for purpose in life, and 0.86 for Self-acceptance.

### 2.5. Statistical Analysis

Results are reported as mean and SEM (Standard Error of Mean) or as frequencies and percentages. An unpaired *t*-test was used to compare groups for age. As regards RESE, PWBS, and Satisfaction With Life Scale scores, given that the assumptions of parametric statistics were not satisfied, nonparametric tests were used. Specifically, the Wilcoxon test was used to compare total or dimensional (for the RESE, PWBS, and Satisfaction With Life Scale) scores obtained in the pre- and postintervention for each group (intervention and control). Comparisons between groups (intervention versus control), at pre- and postintervention, were performed with the Mann-Whitney test. Differences between total scores achieved at baseline by students who completed the postintervention assessment versus students lost during the study (who did not complete the postintervention assessment) were tested with the Mann-Whitney test. Differences among percentages were evaluated by the Chi-square test.

A value of *p* < 0.01 was considered to be indicative of a significant difference.

The posttest effect sizes (based on the difference between the two groups at the posttest) for all outcomes measured (whether statistically significant or not) were calculated using Hedges' g (Cohen's d bias corrected for unequal sample sizes) [35].

We initially examined whether gender and age influenced the levels of effect sizes, even though we were, in any case, reassured by the fact that the experimental and control groups appeared to be well balanced for gender and age. For each outcome variable, we conducted separate analyses to calculate the effect sizes among males and among females. Regarding age, we used the median age to split the sample in order to examine the effect sizes on younger (< or = 15 years) and older (>15 years) students.

Careful examination of the results revealed that the effects of the outcome variables were not significantly associated with either age or gender. Therefore, we proceeded to calculate an overall effect size for all the students, regardless of their age and gender.

All statistical analyses were performed using STATA software version 11.2 (STATA Corporation, College Station, TX, USA).

## 3. Results

The baseline characteristics of participating students who completed the postintervention assessment and those lost to postintervention assessment are shown in Table 1.

It can be seen that, with the exception of self-efficacy, there were no differences between groups at baseline in regulating the negative emotion score which was lower in the students who completed the postintervention compared to those who did not. We also compared the baseline characteristics of those students who remained in the study and completed the postintervention assessment in the intervention and control groups. A separate analysis was then undertaken for those who did not complete the postintervention assessment.

There were few differences between the student groups who remained in the study. Specifically, the mean age was slightly higher in the control group than in the intervention group (control group 15.3 ± 0.09; intervention group 15.0 ± 0.07; *p* = 0.0065). Moreover, the autonomy score was also higher in the control group than in the intervention group (Table 2).

Among those who failed to complete the postintervention assessment, there were no significant differences between intervention versus control groups on any of the baseline demographic or outcome variables.

### 3.1. Differences in the Intervention Group and in the Control Group over Time

The outcome scores of students who completed the postintervention assessment are summarized in Table 2. Self-efficacy in regulating negative emotions improved in the intervention group, although not significantly, whereas no change was observed in the control group. No change was observed in both intervention and control groups with regard to self-efficacy in expressing positive emotions. The intervention was associated with a significant improvement in the overall psychological well-being and in life satisfaction scores. These improvements were not observed in the control group. As for psychological well-being dimensions, the intervention was associated with a significant improvement in environmental mastery and self-acceptance. Purpose in life also improved in the intervention group, but the difference was not statistically significant.

As mentioned, at baseline, the difference between groups was significant regarding autonomy with higher scores in the control group. At postintervention, the same trend was observed; the scores improved for both groups and the difference between the groups was significant.

As regard the effect sizes, we initially examined if gender or age influenced the levels of effect sizes, although we were reassured by the fact that the experimental and control groups appeared well balanced for gender and age. For each outcome variable, we analysed the effect size in a linear regression model in which the effect size was the dependent variable and gender and age were included as covariates. Careful examination of the results revealed that the effects of the assessment variables were not significantly associated with neither age or gender. Therefore, we proceeded to calculate an overall effect size for all the students, regardless of their age and gender. The overall effect sizes varied from very small to small, between 0.01 and 0.40 (Table 2).

## 4. Discussion and Conclusion

An important finding of the current study is that classroom students and school staff conducted the programme effectively. It has been observed that interventions are unlikely to have much practical utility or gain acceptance unless they are feasible and sustainable under real-world conditions [36]. This study shows that the programme can be incorporated into routine educational practices although we concede that the schools participating in the study were particularly motivated, which may limit the generalizability to other schools which are not so motivated. Concerning the implementation costs of the programme, we presume that it is potentially cost-effective because it requires few resources; that is, it requires no outside personnel and can be carried out during regular school hours.

In Italy, to the best of our knowledge, no such long-term structured programmes aimed at promoting mental health, as part of curricular school activities, have been put into action [36] except for a school-based shorter intervention programme which was derived from the well-being therapy and which was tested in a population of high school students [17] with promising clinical results.

To develop the programme, we adopted an approach based on the psychoeducational model promoted mainly in Italy by Falloon for patients with severe mental disorders who are attending psychiatric rehabilitation services. Therefore, it greatly emphasises structured problem-solving techniques. However, in addition, the programme places a high level of importance on defining personal goals and using communication skills [25]. These last two skills make the approach significant and innovative, because promoting students' active involvement in taking decisions concerning their individual objectives could give them greater control over their lives and better personal and social functioning.

In the present study, the effectiveness of the programme was evaluated in a sample of 308 students by means of a pre-post test study design. Findings show that following the programme produced an increase in psychological well-being (particularly in its dimensions of environmental mastery, self-acceptance, and autonomy) and satisfaction with life. Regarding self-efficacy, we observed an increase that did not reach statistical significance. Maybe learning self-efficacy skills needs more intensive and continued application and exercise.

Our results add to a growing body of positive results reported by other authors who have conducted studies which examine programmes aimed at promoting youth development. Extensive research during the past three decades has demonstrated that, over the years, many programmes that teach personal and social competencies such as self-control and stress management have produced positive outcomes in relation to children's and adolescents' social adjustment, assertive behaviour, and coping with stressors [37].

There are some reasons why the programme could play a role in reducing risk factors and incidence of common mental disorders in adolescence. In fact, recently, some studies have demonstrated that improvements in psychological well-being may reduce distress and improve resilience to common mental disorders [17]. The hypothesis is that promoting the emotional strength of an individual, such as psychological well-being and life satisfaction, could act as a protective factor, in particular against depression. This also concords with cognitive theories that posit negative views of the self, the world, and the future as fundamental aspects which characterize depression [38]. However, it remains to be determined whether or not the present programme is truly effective in terms of preventing mental health disorders such as depressive and anxiety disorders.

This study has a number of strengths: pre-post test study design with a control group, anonymous ratings, and standardized measures. Moreover, it evaluated the effectiveness of a structured approach in ordinary schools, an approach that has the potential to reach larger population groups with few professional and financial resources.

The study has also a number of limitations. The optimal sample size was not estimated a priori, and the follow-up period should have been extended. The current follow-up period was only 2 months, due to the preliminary nature of the study, and the observed improvements might be lost over a longer follow-up period. It should be noted, however, that previous studies where outcomes were lost reported a reduction in improvement fairly soon after the end of the prevention programmes [39, 40].

The study had a relatively high student attrition rate, because the study design stated that the students had to be present in class when the questionnaires were administered, and therefore, temporary absence may have affected the response rates during the postintervention assessment. However, among those who failed to participate in postintervention assessments, there were no significant differences between intervention versus control groups for any of the baseline demographic or outcome variables investigated in the study. This indicates that attrition was unlikely to have biased the comparison between programme and control groups.

Another limitation is that differences between pre-and postprogramme scores are small in absolute values. Thus, there were small, albeit statistically significant, effects sizes on a number of scales which assessed psychological well-being and satisfaction with life. An introduction of contamination within the schools, between intervention and control classes, may have played a role in this, weakening or diluting the intervention effects. The control classes within the schools may have become aware of the intervention implementation processes by knowledge transfer from the intervention classes, either inadvertently or intentionally as students may have discussed their experiences. Nevertheless, the programme performance in the intervention group is encouraging and provides preliminary evidence of its efficacy.

We think that future studies in which students practice assiduously and apply targeted skills outside the classroom should obtain better outcomes. This is an important issue because in this programme regular practice is a key component of effective skills acquisition. Moreover, communication skills and problem-solving techniques need to be repeated because they are unlikely to be achieved during only one school year; they demand practice and exercise over time.

In conclusion, these preliminary results suggest that the programme produced significant positive effects on targeted emotional competencies and attitudes about the self in adolescents. Further programme evaluations with longer follow-up may disclose whether the effects become stronger the longer the students are exposed to the programme, and if the programme could truly play a role in preventing stress-related disorders and in promoting successful adaptation.

## Figures and Tables

**Box 1:**
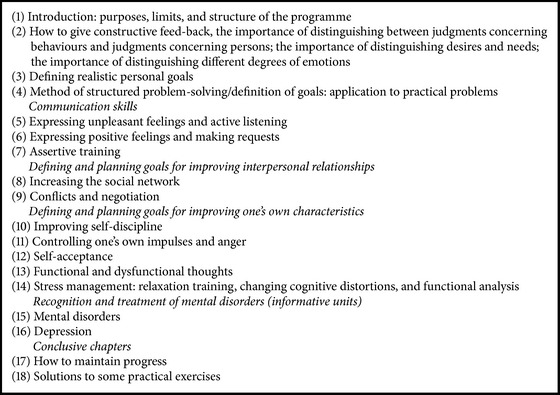
**Box 1: **Units of the student manual for mental-health promotion.

**Table 1:** Comparison between students who completed the postintervention assessment and students that failed to participate in postintervention (*N* = 391).

Variable	Students who completed postintervention assessment (*N* = 308) mean (SEM)	Students not undergoing postintervention (*N* = 83) mean (SEM)	*p* value
Age	15.2 (0.06)	15.5 (0.12)	***0.02*** ^*∗*^
Gender: male (%)	37.3	47.0	*0.11^*∗∗*^*
Self-efficacy in regulating negative emotions	23.1 (0.30)	25.1 (0.63)	***0.00*** ^*∗∗∗*^
Self-efficacy in expressing positive emotions	30.2 (0.26)	29.2 (0.51)	*0.05^*∗∗∗*^*
Satisfaction with life	24.2 (0.32)	23.6 (0.67)	*0.57^*∗∗∗*^*
Psychological well-being	357.9 (2.58)	357.2 (4.94)	*0.88^*∗∗∗*^*

^*∗*^ *t*-test for unpaired data; ^*∗∗*^Chi-square test; ^*∗∗∗*^Mann-Whitney test.

**Table 2:** Mean outcome scores and Standard Error of the Mean (SEM) of participating students (*N* = 308).

Variable	Group	*N*	Baseline mean (SEM) *p* value	Postintervention mean (SEM) *p* value	*p* value	Posttest Effect size
Self-efficacy in regulating negative emotions	Intervention	173	23.0 (0.40)	23.9 (0.44)	*0.0313 *	
	Control	132	23.1 (0.47)	23.4 (0.43)	*0.8868 *	
			*0.8551 *	*0.9221 *		*0.09 *

Self-efficacy in expressing positive emotions	Intervention	173	29.6 (0.36)	29.9 (0.33)	*0.8790 *	
	Control	132	30.9 (0.35)	30.2 (0.46)	*0.6357 *	
			*0.0277 *	*0.0861 *		*0.07 *

Satisfaction with life	Intervention	176	24.2 (0.39)	25.4 (0.37)	***0.0002*** ^**∗∗**^	
	Control	132	24.3 (0.55)	24.8 (0.50)	*0.3098 *	
			*0.3700 *	*0.6446 *		*0.10 *

Psychological well-being (total score)	Intervention	167	355.7 (3.37)	361.4 (3.26)	***0.0148*** ^**∗∗**^	
	Control	129	361.5 (4.03)	365.4 (4.25)	*0.4059 *	
			*0.1101 *	*0.1524 *		*0.09 *

Autonomy	Intervention	167	57.7 (0.89)	58.1 (0.76)	*0.1502 *	
	Control	129	60.7 (0.99)	62.2 (0.93)	*0.0483 *	
			***0.0069*** ^**∗**^	***0.0006*** ^**∗**^		*0.40 *

Environmental mastery	Intervention	167	57.6 (0.73)	59.0 (0.69)	***0.0157*** ^**∗∗**^	
	Control	129	58.6 (0.84)	59.0 (0.84)	*0.9782 *	
			*0.1299 *	*0.7459 *		*0.01 *

Personal growth	Intervention	167	61.5 (0.57)	62.0 (0.59)	*0.2601 *	
	Control	129	60.5 (0.69)	61.2 (0.79)	*0.1397 *	
			*0.6679 *	*0.9794 *		*0.10 *

Positive relations with others	Intervention	167	64.5 (0.83)	64.3 (0.84)	*0.5221 *	
	Control	129	65.2 (0.93)	64.7 (0.95)	*0.2848 *	
			*0.3979 *	*0.6879 *		*0.04 *

Purpose in life	Intervention	167	57.5 (0.77)	58.7 (0.73)	*0.0612 *	
	Control	129	58.1 (0.83)	59.3 (0.88)	*0.1982 *	
			*0.4140 *	*0.4465 *		*0.07 *

Self-acceptance	Intervention	167	56.9 (0.93)	59.4 (0.89)	***0.0015*** ^**∗∗**^	
	Control	129	58.3 (1.18)	59.0 (1.04)	*0.8026 *	
			*0.1173 *	*0.9417 *		*0.03 *

^*∗*^Mann-Whitney test; ^*∗∗*^Wilcoxon test.
